# Mesenchymal Remodeling during Palatal Shelf Elevation Revealed by Extracellular Matrix and F-Actin Expression Patterns

**DOI:** 10.3389/fphys.2016.00392

**Published:** 2016-09-07

**Authors:** Matthias Chiquet, Susan Blumer, Manuela Angelini, Thimios A. Mitsiadis, Christos Katsaros

**Affiliations:** ^1^Department of Orthodontics and Dentofacial Orthopedics, Medical Faculty, School of Dental Medicine, University of BernBern, Switzerland; ^2^Orofacial Development and Regeneration, Center for Dental Medicine, Institute for Oral Biology, University of ZurichZurich, Switzerland

**Keywords:** palate morphogenesis, palatal shelf elevation, extracellular matrix, actin, tissue remodeling, mouse embryo

## Abstract

During formation of the secondary palate in mammalian embryos, two vertically oriented palatal shelves rapidly elevate into a horizontal position above the tongue, meet at the midline, and fuse to form a single entity. Previous observations suggested that elevation occurs by a simple 90° rotation of the palatal shelves. More recent findings showed that the presumptive midline epithelial cells are not located at the tips of palatal shelves before elevation, but mostly toward their medial/lingual part. This implied extensive tissue remodeling during shelf elevation. Nevertheless, it is still not known how the shelf mesenchyme reorganizes during this process, and what mechanism drives it. To address this question, we mapped the distinct and restricted expression domains of certain extracellular matrix components within the developing palatal shelves. This procedure allowed to monitor movements of entire mesenchymal regions relative to each other during shelf elevation. Consistent with previous notions, our results confirm a flipping movement of the palatal shelves anteriorly, whereas extensive mesenchymal reorganization is observed more posteriorly. There, the entire lingual portion of the vertical shelves moves close to the midline after elevation, whereas the mesenchyme at the original tip of the shelves ends up ventrolaterally. Moreover, we observed that the mesenchymal cells of elevating palatal shelves substantially align their actin cytoskeleton, their extracellular matrix, and their nuclei in a ventral to medial direction. This indicates that, like in other morphogenetic processes, actin-dependent cell contractility is a major driving force for mesenchymal tissue remodeling during palatogenesis.

## Introduction

The secondary palate, which separates the oral cavity from the nasal cavity, is a specific feature of mammals (Ferguson, 1988). Cleft lip/palate is the most frequent craniofacial malformation in humans (Jugessur et al., 2009a; Mossey et al., 2009; Dixon et al., 2011). Formation of the secondary palate during embryogenesis is a complex process involving extensive tissue growth and remodeling (Gritli-Linde, 2008; Jugessur et al., 2009b; Bush and Jiang, 2012). Palate development starts from the palatal shelves, extensions of the maxillary processes that are located left and right from the tongue. The palatal shelves initially have a vertical orientation, but around the 6th week of gestation in humans, or at embryonic day 14.5 (E14.5) in mice, they elevate rapidly into a horizontal position above the tongue. The shelves meet medially, and fuse by disappearance of the midline epithelium and merging of the mesenchyme (Gritli-Linde, 2007; Bush and Jiang, 2012). While much information exists concerning the mechanism of palatal shelf fusion (Kaartinen et al., 1995; Proetzel et al., 1995) (Jin et al., 2014), the process of shelf elevation still remains elusive, and knowledge about its cellular and molecular basis is scarce (Bush and Jiang, 2012).

Palatal shelves are known to be divided into distinct regions along their anterior-posterior axis. Their frontal and medial parts will give rise to the hard palate, their posterior part to the soft palate (Yu and Ornitz, 2011; Smith et al., 2012). It was debated whether palatal shelves elevate and fuse first from the front or from the back (reviewed in Brinkley and Vickerman, 1979). *In vitro*, their medial parts elevate first (Brinkley and Vickerman, 1979), whereas in the embryo, tongue movements strongly affect shelf elevation (Iseki et al., 2007; Kouskoura et al., 2016). This causes a remarkable variability in the time course of elevation *in vivo* between siblings, and even between the two shelves of one embryo (Luke, 1984; Iseki et al., 2007; Yu and Ornitz, 2011). A recent histomorphometric analysis concludes that in the embryo, elevation usually starts at the medial to posterior part of the palatal shelves, which then zipper in both directions (Yu and Ornitz, 2011).

The above findings imply that all parts of the palatal shelves have an intrinsic capability to elevate. However, it has been debated how this reshaping occurs, and several reports indicated regional differences in tissue reorganization during shelf elevation. Histological observations suggested that anteriorly, the palatal shelves perform a simple flipping movement (Coleman, 1965). However, other reports indicated that the medial and posterior parts of the shelves remodel extensively during elevation, such that their lingual side protrudes whereas the ventral tip regresses (Brinkley and Vickerman, 1979; Chou et al., 2004). A recent study used the expression of the *Mmp13* gene to determine the origin of midline epithelial cells during palatal shelf elevation. Its results supported a flipping movement of the shelves in the front and extensive remodeling posteriorly (Jin et al., 2010), which was confirmed by careful histomorphometric analysis (Yu and Ornitz, 2011).

Although there is general agreement that extensive remodeling of the palatal shelves occurs during elevation, it is not known how different regions of the palatal mesenchyme move relative to each other. Moreover, the forces that drive tissue remodeling in the elevating shelves are still unknown. Distinct patterns of cell proliferation (Sasaki et al., 2004) and density (Brinkley and Bookstein, 1986) are unlikely to contribute substantially to the remodeling process, since it occurs in less than 1 day in mouse embryos (Yu and Ornitz, 2011). The finding of hyaluronidase-sensitive material in the “hinge” region of palatal shelves led to the hypothesis that rapid accumulation of hyaluronic acid or other glycosaminoglycans might provide the driving force for shelf elevation, by generating osmotic swelling pressure (Brinkley and Morris-Wiman, 1987). Alternatively, this process might be powered by coordinated cytoskeletal contractions of both epithelium and mesenchyme, similar to what is observed in many other morphogenetic tissue rearrangements (Wozniak and Chen, 2009). Although an early report suggested a role for actin-based contractility in palatal shelf elevation (Lessard et al., 1974), to our knowledge this idea has never been followed up.

Therefore, in the present study we address two important questions concerning the process of palatal shelf elevation. Firstly, is it possible to detect rearrangements of different parts of the palatal mesenchyme relative to each other during the elevation process? Secondly, can we find any evidence for actin-based contractility in the remodeling of palatal mesenchyme during elevation? To address these points, we took advantage of the fact that certain extracellular matrix (ECM) components have a restricted expression in defined regions of the palatal mesenchyme, which can be followed during shelf elevation. Furthermore, we stained sections through elevating palatal shelves for F-actin, and detected elongated cell nuclei that aligned with the actin network. Our results confirm and refine previous findings of extensive remodeling of the palatal mesenchyme at medial and posterior levels, and provide the first evidence for tensile stress acting within this tissue during the process of shelf elevation.

## Materials and methods

### Animals, embryonic tissues, and cryosectioning

C57BL/6 wild-type mouse embryos were obtained from J.-F. Spetz at the Friedrich-Miescher Institute for Biomedical Research in Basel, Switzerland, or from in house breeding at the central animal facilities, Department of Clinical Research, University of Bern. After mating, appearance of a vaginal plug was considered embryonic day 0.5 (E0.5). Pregnant females were sacrificed at the desired stage (E13.5–E14.5), embryos were removed from the uterus and decapitated. Animal experiments were approved by the Cantonal Veterinary Offices of Basel, Zurich and Bern, Switzerland. The embryo heads were washed in ice-cold PBS, fixed in 4% paraformaldehyde in phosphate buffered saline (PBS; 150 mM NaCl, 20 mM Na-phosphate, pH 7.4) overnight, soaked for 24 h in 30% sucrose in PBS, embedded in Tissue Tek (O.C.T. compound; Sakura Finetek Europe B.V., Zoeterwoude, Netherlands), and frozen on an aluminum block cooled to −80°C. Frozen heads were stored at −80°C before sectioning. Serial frontal sections (12 μm thick) were prepared on a Cryocut E cryomicrotome (Reichert-Jung, Leica Microsystems, Heerbrugg, Switzerland). Sections were dried at 37°C for 1–5 min, and stored at −80°C for further use.

### Gene-specific RNA probes and *in situ* hybridization

Total RNA was isolated from E14.5 C57BL/6 wildtype mouse embryos using an RNAeasy Mini Kit (Qiagen, Hombrechtikon, Switzerland), and reverse transcribed to cDNA using Moloney murine leukemia virus reverse transcriptase (Promega, Dübendorf, Switzerland). Gene-specific primers (Microsynth, Balgrach, Switzerland) were designed using free NCBI software (http://www.ncbi.nlm.nih.gov/tools/primer-blast/index.cgi?LINK_LOC=BlastHome). Primers were fitted with BamH1 (forward primers) or HindIII (reverse primers) restriction sites at their 5′ ends, respectively. With these primers (Table 1), specific products were amplified from E14.5 mouse cDNA by PCR using Go Taq polymerase (Promega), cut with respective restriction enzymes, and cloned into pBluescript SK+ plasmid (Stratagene/Agilent, Santa Clara, USA). In the case of mouse *tenascin-C* (*Tnc*) and *tenascin-W* (gene name *Tnn*), full-length cDNA clones were obtained from R. Chiquet-Ehrismann (Friedrich Miescher Institute for Biomedical Research, Basel, Switzerland), and restriction fragments of suitable size (Table 1) were cloned into pBluescript SK+. Plasmids were linearized by cutting upstream or downstream of the insert, respectively, and digoxygenin-labeled anti-sense or sense RNA probes were synthesized (Koch et al., 1995) with a labeling kit from Roche Diagnostics using T3 or T7 RNA polymerase (Promega). *In situ* hybridization was done with the labeled probes as published in detail before (Flück et al., 2000). After incubation with alkaline phosphatase conjugated anti-digoxygenin antibody (Roche Diagnostics), a color substrate solution was used to which 10% polyvinyl alcohol (MW 31′000-50′000; Sigma-Aldrich, Buchs, Switzerland) was added (Shen, 2001). After development, the sections were counterstained with Nuclear Fast Red (Sigma).

**Table 1:** **Gene-specific RNA probes for ***in situ*** hybridization**.

**MOUSE PERIOSTIN (Postn: NM_015784.3)**
Forward primer: 5′CCGGATCCGCAGCCGCCATCACCTCTGAC 3′ (nucleotide: 196-216)
Reverse primer: 5′ CCAAGCTTGCTTGTCTTGCCGCAGCTTGT 3′ (nucleotide: 964-944)
PCR product/probe length (without restriction sites): 769 bp
**MOUSE TENASCIN-C (Tnc: D_90343.1)**
Restriction fragment: Sac1 (nucleotide 5862)–EcoR1 (nucleotide 6813)
Probe length: 950 bp
**MOUSE TENASCIN-W (Tnn: AJ_580920.1)**
Restriction fragment: HindIII (nucleotide 1190)–BamH1 (nucleotide 1440)
Probe length: 250 bp
**MOUSE SMOC-2 (Smoc2: NM_022315.2)**
Forward: 5′ CCGGATCCCAGGTGCCCCGGTTCAA 3′ (nucleotide: 661-679)
Reverse: 5′ CCAAGCTTCCAGGGTGTGGCTGGGGT 3′ (nucleotide: 1253-1235)
PCR product/probe length (without restriction sites): 593 bp
**MOUSE TRANSFORMING GROWTH FACTOR-**β**3 (Tgfb3: NM 009368.3)**
Forward primer: 5′ CCGGATCCCAACCCCAGCTCCAAGCG 3′ (nucleotide: 1578-1597)
Reverse primer: 5′ CCAAGCTTCCAGGTTGCGGAAGCAGT 3′ (nucleotide: 2046-2026)
PCR product/probe length (without restriction sites): 469 bp

### Fluorescence labeling of tissue sections

Monoclonal antibody mTn12 against mouse tenascin-C (Aufderheide and Ekblom, 1988) was obtained from R. Chiquet-Ehrismann (Friedrich Miescher Institute for Biomedical Research, Basel, Switzerland). Cryosections from paraformaldehyde-fixed mouse embryo heads were blocked with 3% bovine serum albumin in phosphate-buffered saline (BSA/PBS) and incubated with anti-tenascin antibody (1:100 in BSA/PBS) for 1 h at room temperature. After washing with PBS containing 0.2% Tween-20, sections were incubated with Alexa488-labeled secondary antibody (Invitrogen; 1:1000 in BSA/PBS). For labeling the F-actin network on the same section, 1 μg/ml rhodamine-phalloidin (Sigma) was added to the secondary antibody solution. After washing again with PBS/Tween, sections were embedded in phosphate-buffered glycerol (pH 7.4) containing 1 μg/ml DAPI stain (Sigma) to label nuclei.

### Microscopy

Slides processed for *in situ* hybridization were viewed under bright field optics with 4x or 10x objectives on an Olympus BX-51 microscope. Slides triple-labeled with fluorescent dyes were observed on the same microscope by epifluorescence optics, using 10x and 20x fluorescence objectives and the following Olympus filter/mirror series: U-MWIBA3 for Alexa488; U-MWIGA3 for rhodamine; U-MNUA2 for DAPI. Digital images were recorded using a ProgRes CT3 CMOS camera and ProgRes Capture Pro software (Jenoptik, Jena, Germany). Slides from one experiment were photographed at identical camera settings, and resulting images were processed identically.

### Image analysis

DAPI-labeled nuclei on tissue sections were color-coded depending on their aspect ratio (ratio of x to y axis) in the following way. Images were imported into ImageJ, binarized, and the Watershed tool was used to separate touching/overlapping nuclei. Particles (i.e., DAPI-stained nuclei) were fitted with ellipses, and their aspect ratio was analyzed. A ROI (region of interest) color coder plugin with the “Phase” LUT (look up table) was then used to color-code all nuclei with an aspect ratio of >2.5 and above in red, 2.0–2.5 in pink, and < 2.0 in blue.

## Results

### Periostin as a mesenchymal marker for palatal shelves during elevation

To generate a fate map of palatal mesenchyme and epithelium during the process of shelf elevation, we screened the expression patterns of more than 20 ECM components by *in situ* hybridization on frontal sections of wild-type C56/BalbC mouse embryo heads during the relevant stages, i.e., E13.5–E14.5. Of these genes, the expression of eight was highly enriched in either all or just a specific region of the palatal shelves compared to the surrounding maxillary tissue: Periostin, tenascin-C, tenascin-W, collagen types II, IX, XI, and XIV, and SMOC-2.

**Figure 1:**
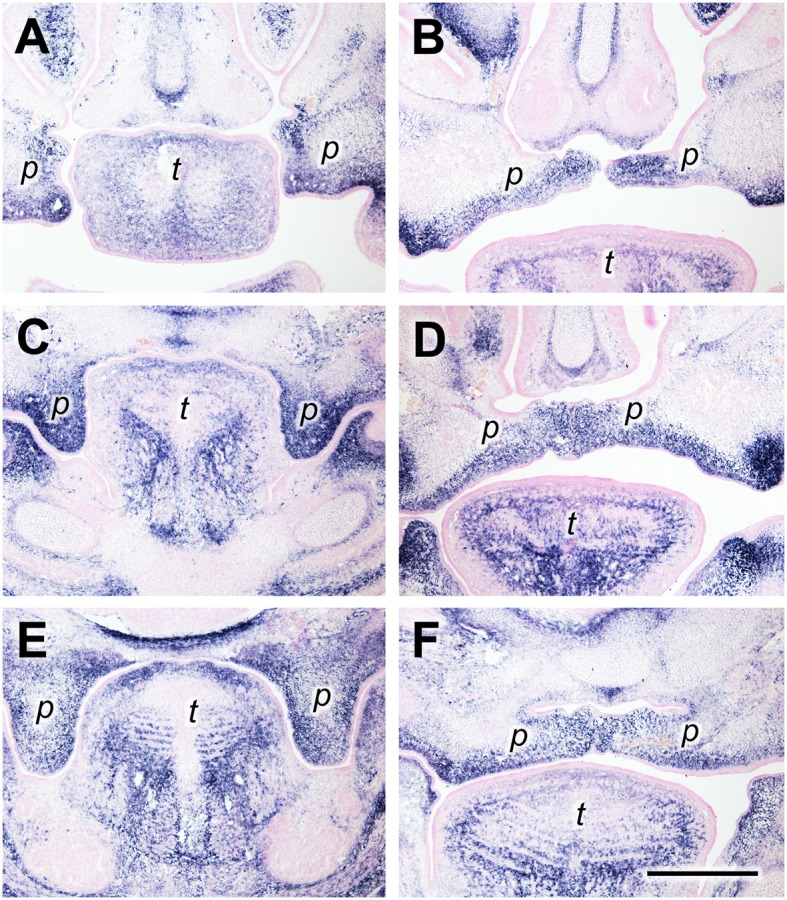
*****Periostin*** expression as a marker for palatal shelves during elevation**. *In situ* hybridization for *Postn* mRNA on frontal sections of palatal shelves from wild-type mouse embryos at the anterior **(A,B)**, medial **(C,D)**, and posterior **(E,F)** level. Serial sections were obtained from an E13.5 **(A,C,E)** and an E14.5 **(B,D,F)** embryo, respectively. For description, see text. Abbreviations: palatal shelf (p), tongue (t). Bar, 400 μm.

Periostin (gene name *Postn*) is an ECM component belonging to the “matricellular” proteins (Murphy-Ullrich and Sage, 2014) and known to be involved in morphogenesis and wound healing (Walker et al., 2016). Periostin protein was demonstrated in mouse palatal shelves by immunofluorescence (Oka et al., 2012). Here, we used *in situ* hybridization to reveal the expression pattern of *Postn* mRNA in palatal shelves of wild-type mouse embryos immediately before (E13.5) and after (E14.5) elevation. Serial frontal cryosections through palatal shelves were hybridized with *Postn*-specific RNA probe at three levels: anterior (future anterior hard palate), medial (future posterior hard palate), and posterior (future soft palate). Anteriorly, *Postn* mRNA was expressed in a broad layer of mesenchyme on both the buccal (ventrolateral) and lingual (medial) aspect of E13.5 palatal shelves (Figure 1A). After shelf elevation (E14.5), the layer of *Postn* expression was located ventrolaterally in the shelves, and filled their medial tips (Figure 1B). At the medial level, a strong *Postn* signal covered the entire mesenchymal area of vertical palatal shelves at E13.5 (Figure 1C). After elevation at this level, *Postn* expression was observed in a broad area around the midline, from where it extended into a ventral stripe facing the oral cavity (Figure 1D). In contrast and remarkably, the dorsolateral regions of the elevated shelves were free of *Postn* signal (Figure 1D). A similar distribution was seen at the posterior level both before and after elevation, except that the signal was weaker in the central mesenchyme region of the shelves (Figures 1E,F).

### Tenascin-C expression in the medial palatal mesenchyme both before an after shelf elevation

**Figure 2:**
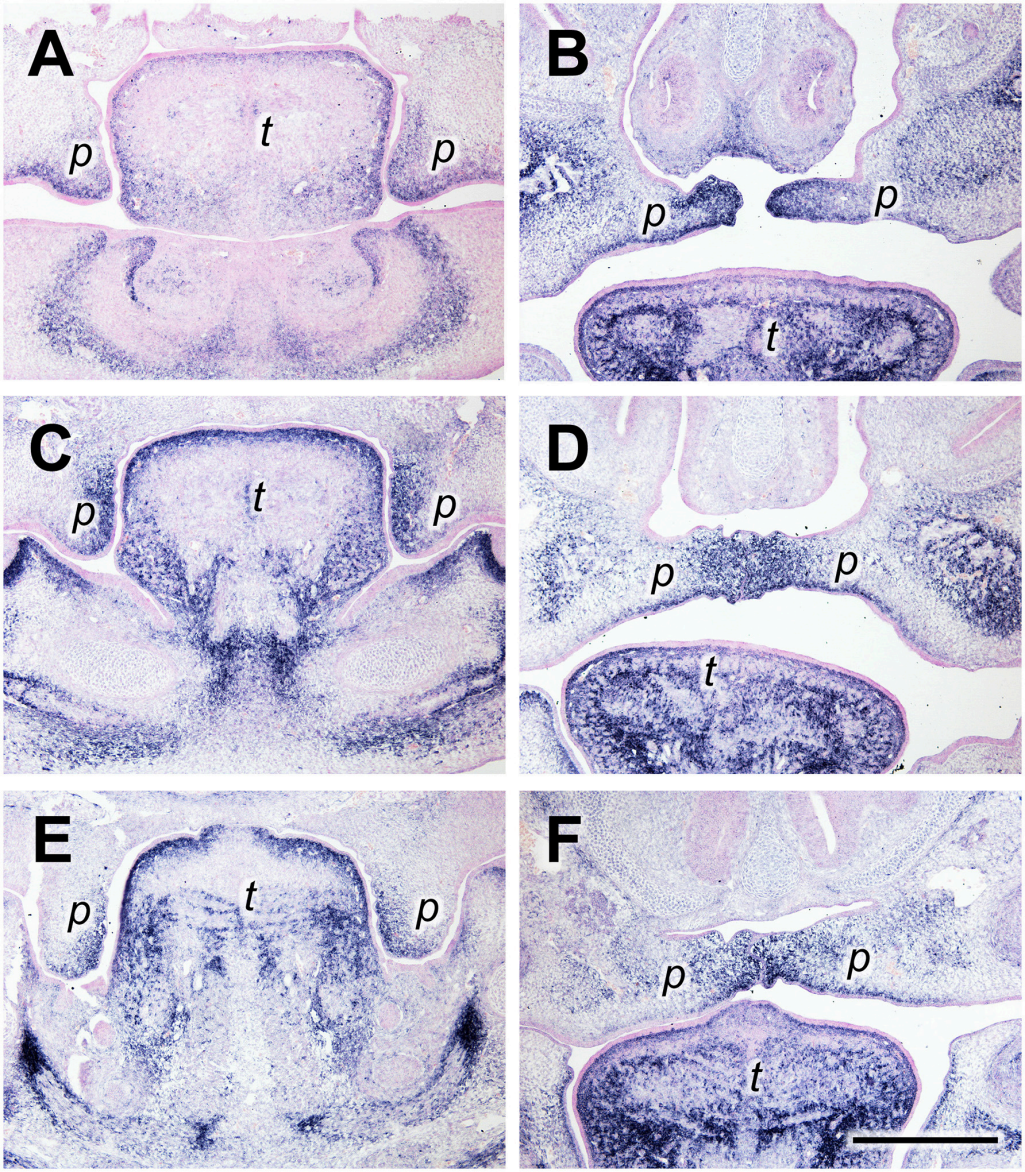
*****Tenascin-C*** expression primarily in the lingual/medial half of palatal shelves both before and after shelf elevation**. *In situ* hybridization for *Tnc* mRNA on frontal sections of palatal shelves from wild-type mouse embryos at the anterior **(A,B)**, medial **(C,D)**, and posterior **(E,F)** level. Serial sections were obtained from an E13.5 **(A,C,E)** and an E14.5 **(B,D,F)** embryo, respectively. For description, see text. Abbreviations: palatal shelf (p), tongue (t). Bar, 400 μm.

Tenascin-C (gene name *Tnc*) is another “matricellular” ECM protein with highly regulated patterns of expression in vertebrate morphogenesis and pathologies (Chiquet-Ehrismann and Chiquet, 2003). Like periostin, tenascin-C protein has been detected in the developing secondary palate before by immunofluorescence (Ferguson, 1988). At the anterior level at E13.5, the *Tnc* mRNA expression pattern resembled that of periostin: In vertical shelves, the signal was found in the sub-epithelial mesenchyme both on the lingual (medial) and the buccal (ventrolateral) aspect (Figure 2A). After elevation, *Tnc* mRNA was concentrated at the tips of the shelves and extended laterally into the subepithelial mesenchyme both dorsally and ventrally (Figure 2B). A different distribution was observed at the medial level, i.e., in the future posterior hard palate. There, *Tnc* expression covered the entire lingual half of the vertical shelves at E13.5, whereas the buccal half was devoid of signal except for a thin stripe underneath the epithelium (Figure 2C). In elevated shelves (E14.5) at this level, *Tnc* mRNA was observed in a broad mesenchymal region that extended from dorsal to ventral around the midline, and in a thin stripe underneath the ventral epithelium (Figure 2D). A similar distribution was seen for *Tnc* at the posterior level (future soft palate), although the signal was weaker (Figures 2E,F).

### Dorsomedial tenascin-w expression in palatal shelves both before and after shelf elevation

**Figure 3:**
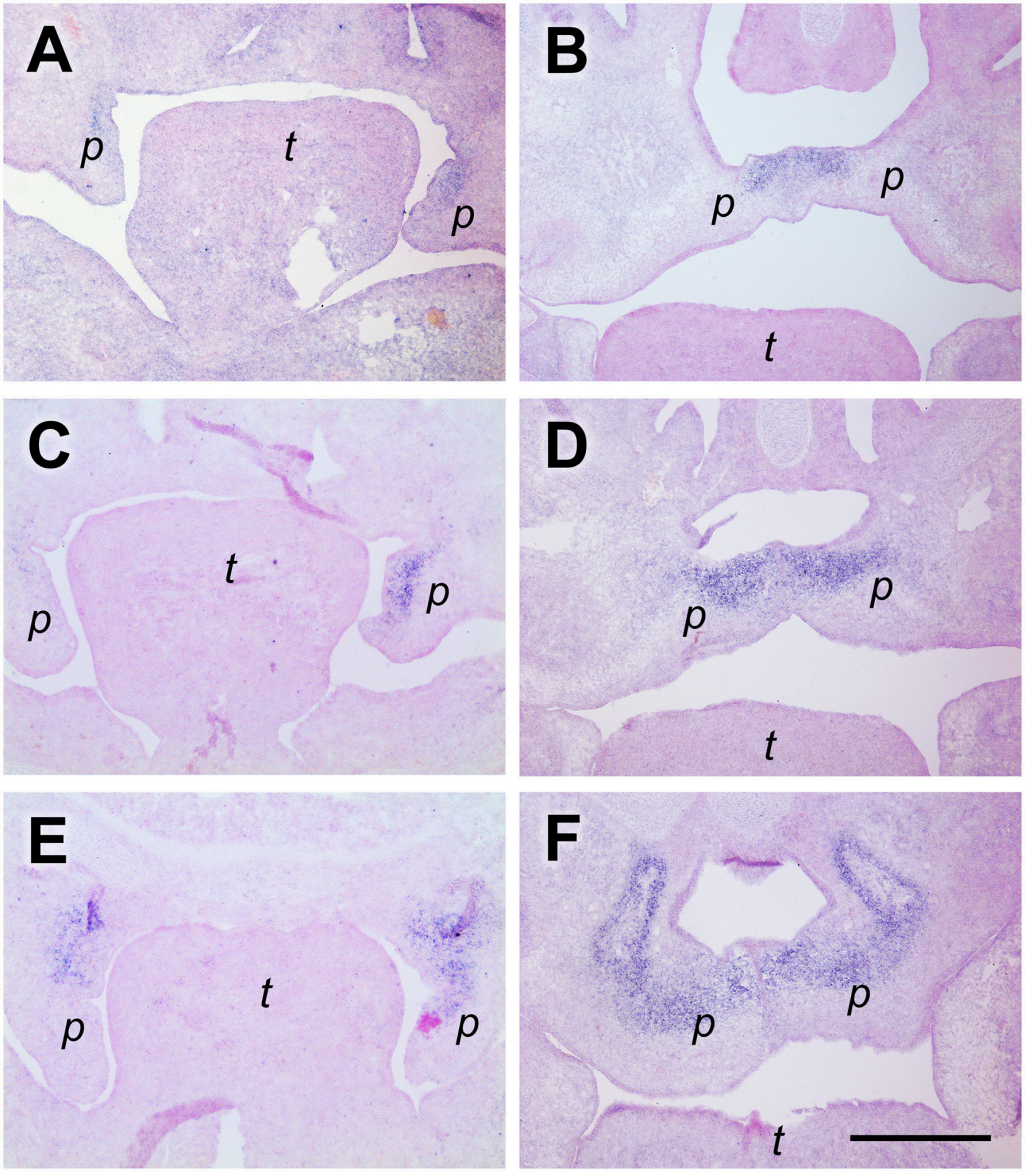
*****Tenascin-W*** expression in the dorsomedial quadrant of palatal shelves both before and after elevation**. *In situ* hybridization for *tenascin-W*/*Tnn* mRNA on frontal sections of palatal shelves from wild-type mouse embryos at the anterior **(A,B)**, medial **(C,D)**, and posterior **(E,F)** level. Serial sections were obtained from an E13.5 **(A,C,E)** and an E14.5 **(B,D,F)** embryo, respectively. For description, see text. Abbreviations: palatal shelf (p), tongue (t). Bar, 400 μm.

Tenascin-W (gene name *Tnn*) is the latest member of this family of ECM proteins (Scherberich et al., 2004). During embryogenesis, its expression partially overlaps with that of tenascin-C but is even more restricted. Notably, tenascin-W was shown to be involved in osteogenesis (Martina et al., 2010) and is a marker for osteogenic areas in embryos (Scherberich et al., 2004). In anterior palatal shelves at E13.5, we found *Tnn* to be expressed exclusively in the dorsomedial quadrant of the mesenchyme, whereas the tips of the vertical shelves were devoid of signal (Figure 3A). At E14.5, the *Tnn* signal was restricted to the dorsal mesenchyme around the midline of the elevated shelves (Figure 3B). A similar distribution was found at the medial level both at E13.5 and E14.5, except that in the elevated shelves the area of *Tnn* expression was spread out laterally and filled the entire dorsal half of the shelf mesenchyme (Figures 3C,D). More posteriorly, the Tnn signal was present in addition around the developing os palatinum (Figures 3E,F).

### SMOC-2 expression at palatal shelf tips before, but not at the midline after elevation

**Figure 4:**
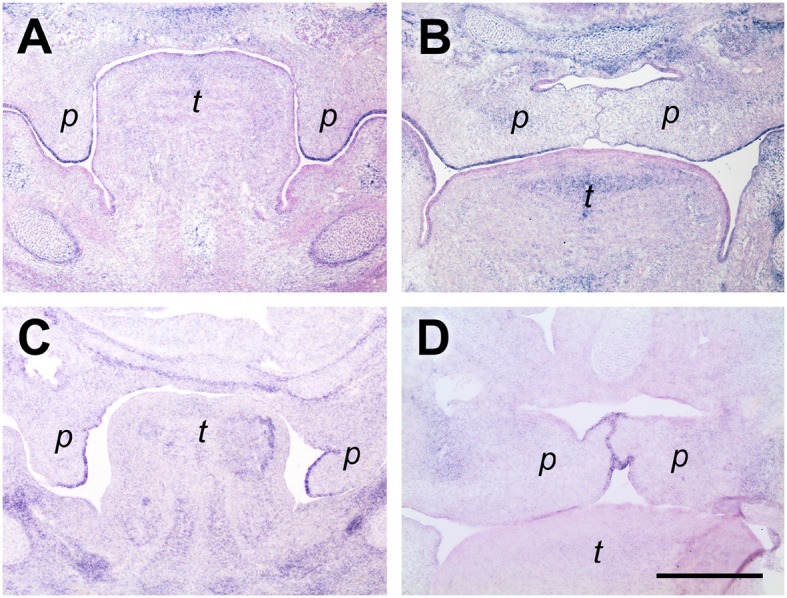
*****Smoc2*** expression in palatal tip epithelium before, but not midline epithelium after elevation**. *In situ* hybridization for *Smoc2* **(A,B)** or *Tgf*β*3* **(C,D)** mRNA, respectively, on frontal sections at the medial level of palatal shelves from wild-type mouse embryos. Serial sections were obtained from an E13.5 **(A,C,E)** and an E14.5 **(B,D,F)** embryo, respectively. For description, see text. Abbreviations: palatal shelf (p), tongue (t). Bar, 400 μm.

SMOC-2 (SPARC related modular calcium binding 2; gene name *Smoc2*) belongs to the SPARC/Osteonectin family of matricellular proteins (Vannahme et al., 2003) and is involved in keratinocyte migration (Maier et al., 2008) and angiogenesis (Rocnik et al., 2006). In mouse embryos, *Smoc2* is prominently expressed in perichondrial mesenchyme (Figure 4A). Surprisingly, *Smoc2* mRNA is also detected in defined areas of the oral epithelium. At a medial to posterior level before elevation (E13.5), the *Smoc2* signal outlines the buccal surface of the palatal shelves and extends into the epithelium at their tips, but finishes abruptly at the lower end of their lingual aspect (Figure 4A). At E14.5, *Smoc2* mRNA is present in the entire ventral epithelium of the elevated shelves, but stops shortly before the midline (Figure 4B). A similar expression pattern in palatal epithelium before and after elevation was found for type XIV collagen, also named undulin, a minor fibril-associated collagen (Ricard-Blum, 2011) (not shown). For comparison, sections from a similar level were hybridized with a *Tgf*β*3* probe. This growth factor is secreted by midline epithelial cells and required for palatal shelf fusion (Kaartinen et al., 1995). At E13.5, *Tgf*β*3* mRNA extended from the shelf tip about halfway up the lingual epithelium (Figure 4C), and at E14.5 was found at the midline (Figure 4D). Thus, *Smoc2* and *Tgfb3* exhibit a reciprocal expression pattern in the palatal epithelium, except for a short overlap at the shelf tip before, or close to the ventral midline after elevation, respectively.

### Mesenchymal and epithelial rearrangements during palatal shelf elevation at the medial level

**Figure 5:**
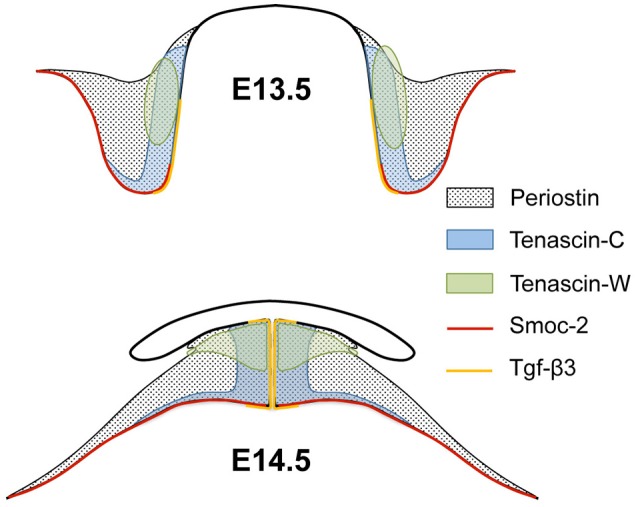
**Scheme of the combined expression patterns of ECM genes in palatal shelves before and after elevation**. The schematic drawings represent palatal shelves of wild-type mouse embryos at the medial level before (E13.5; above) and after (E14.5; below) elevation. The approximate areas of mesenchymal expression of *periostin, tenascin-C, tenascin-W*, and epithelial expression of *Smoc2* and *Tgf*β*3* are indicated. Note that *tenascin-C, tenascin-W*, and *Tgf*β*3* expressing areas all end up at the midline after elevation, although their center of expression does not localize to the shelf tip before elevation. Conversely, *Smoc2* mRNA is epithelially expressed at the shelf tip before, but excluded from midline epithelium after elevation.

Since the mRNAs of the chosen ECM genes were enriched in distinct areas of the palatal shelves before and after elevation, this allowed the mapping of their combined expression patterns (Figure 5; compare with Figures 1–4). At the medial level, the combined results implied that elevation is not due to a simple rotation of the palatal shelves from the vertical into the horizontal plane. If this were the case, the *Tnc*-expressing lingual half of the vertical shelves should end up dorsally in the elevated secondary palate. Instead, the *Tnc*-expressing area had moved to a broad zone at the midline, whereas the lateral parts of the elevated shelves were devoid of *Tnc* signal. The *Postn* signal, which filled the entire shelves at E13.5, was drawn out after elevation into a broad tringle that filled the entire mesenchyme around the midline, and tapered on the ventrolateral aspect of the shelves (cf. Figure 1D). Mesenchyme around the nasopharyngeal fold that forms at E.14.5 lacked expression of *Postn* (Figure 5), suggesting that maxillary tissue originally outside the palatal shelf proper is drawn into the elevated shelves at E14.5. Thus, at this level the palatal shelf bulges out medially whereas the original tip retracts during elevation. This involves massive reorganization of the palatal mesenchyme: The lingual mesenchyme of the E13.5 shelf, rather than that at the distal tip, forms the tissue around the midline at E14.5. The mesenchyme at the original tip ends up more laterally after elevation, whereas the originally buccal mesenchyme distorts and spreads out along the ventrolateral aspect of the elevated shelves. Remodeling of the mesenchyme is paralleled by reorganization of the palatal epithelium. *Smoc2* mRNA was detected in shelf tip epithelium before, but not in midline epithelium after elevation. Conversely, *Tgf*β*3* was expressed primarily in lingual epithelium of shelves before, and in midline epithelium after elevation (Figures 4, 5).

### Anterior to posterior differences in ECM rearrangement during shelf elevation

**Figure 6:**
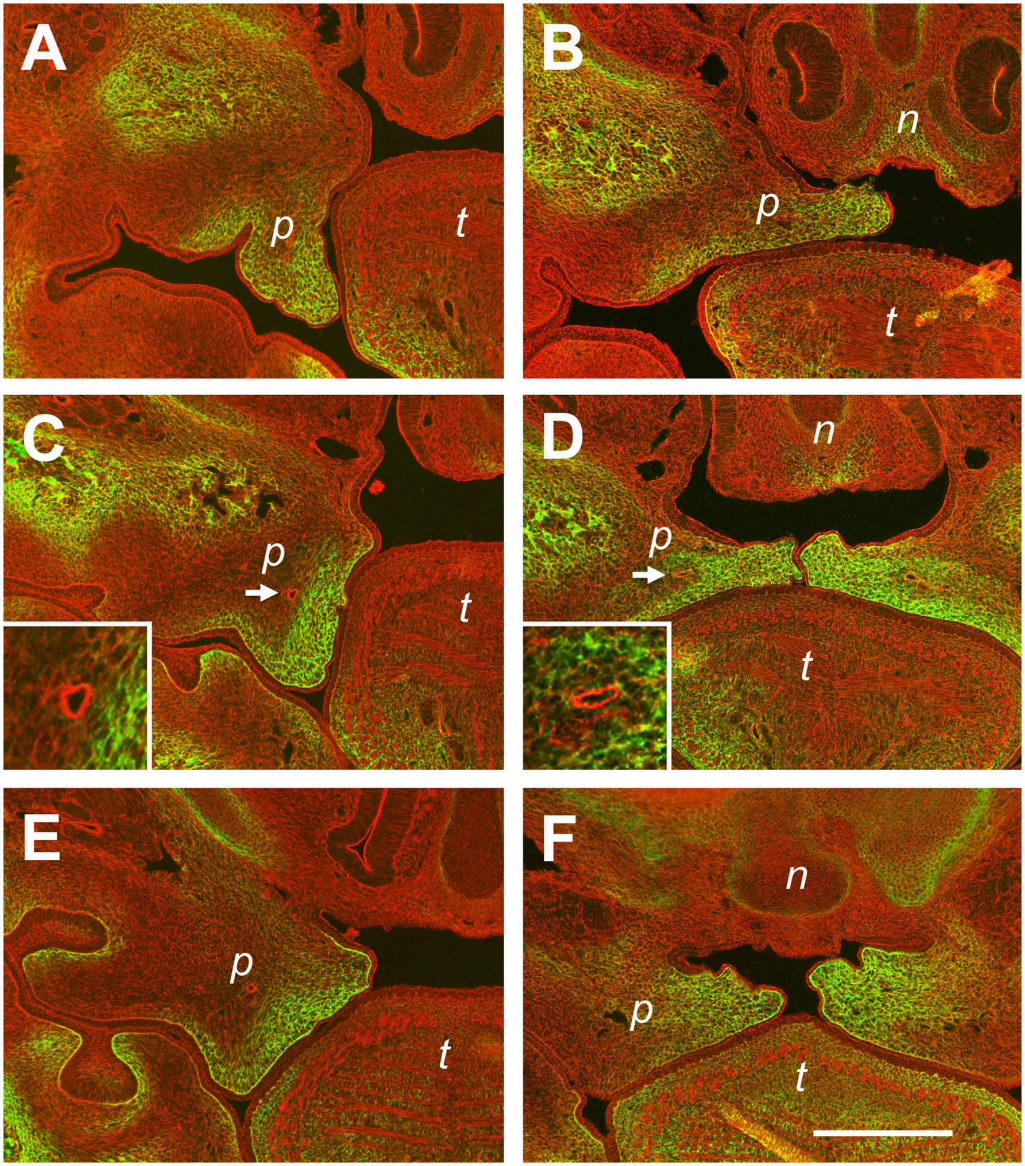
**Differences in tissue rearrangements between anterior, medial, and posterior palatal shelves during elevation**. Serial sections through E14.5 palatal shelves at the anterior **(A,B)**, medial **(C,D)**, and posterior **(E,F)** level were double-stained with rhodamine-phalloidin for actin (red) and antibody to tenascin-C (green), and viewed in a fluorescence microscope. Sections were obtained from two embryos of the same E14.5 litter; one in which the shelves had just started to elevate **(A,C,E)**, and one in which they had already risen above the tongue **(B,D,F)**. Abbreviations: palatal shelf (p), tongue (t), nasal septum (n). The palatine artery is marked with an arrow in **(C,D)**, and shown enlarged in the inserts. Bar, 400 μm.

In wild-type Balb/C litters collected at E14.5, it is quite frequent to find embryos in which either one or both of the palatal shelves have not elevated yet (Luke, 1984; Yu and Ornitz, 2011). These mice can be compared to their siblings with already elevated shelves, and one representative example is shown in Figure 6. The images show frontal sections at three levels through the palatal shelves of two different E14.5 embryos from the same litter, stained by fluorescence for actin (red) and tenascin-C (green). Figure 6A shows a still vertical palatal shelf of one E14.5 embryo at the anterior level, and it is noteworthy that a prominent epithelial invagination has formed on its buccal side. Staining for tenascin-C protein largely fills the shelf mesenchyme, except for its central area. At the same level in the sibling (Figure 6B), its elevated shelf occupies the space between nasal process and tongue, and the epithelial invagination has disappeared. Tenascin-C is present in the medial and ventrolateral parts of the shelf. Since tenascin-C positive regions largely correspond to each other before and after elevation, these observations agree with a flipping movement of the anterior palatal shelves. On the other hand, comparison of shelves from the same two embryos also confirms that extensive mesenchymal reorganization takes place at the medial and posterior level during elevation. At the medial level, a small protrusion has formed on the dorsolingual aspect of the still vertical shelf (Figure 6C). Tenascin-C is found primarily in the lingual half of the shelf mesenchyme, with only a narrow ribbon underneath the buccal epithelium. In case of the elevated shelf at the same level (Figure 6D), the entire tenascin-C containing mesenchymal shelf ECM between the original tip and the dorsolingual protrusion appears compressed between nasal process and tongue. Remarkably, the palatal artery appears circular in cross-section by actin staining before elevation, but is flattened in the vertical direction after elevation (c.f. Figures 6C,D). At the posterior level, the dorsolingual protrusion is very prominent on the not yet elevated shelf, forming a second tip (Figure 6E). Tenascin-C protein is enriched in the mesenchyme between the two shelf tips, rather than at the tips themselves. At this level, it is most obvious that the entire tenascin-C containing mesenchyme has moved to the midline after elevation (Figure 6F). Rather than a flipping movement as seen at the front, this again implies a slug-like reshaping of the medial/posterior palatal shelf, with a deformation and medial movement of the lingual mesenchyme (including the palatine artery) and a retraction of the original shelf tip.

### Orientation of actin fibers and nuclei in palatal mesenchyme during elevation

**Figure 7:**
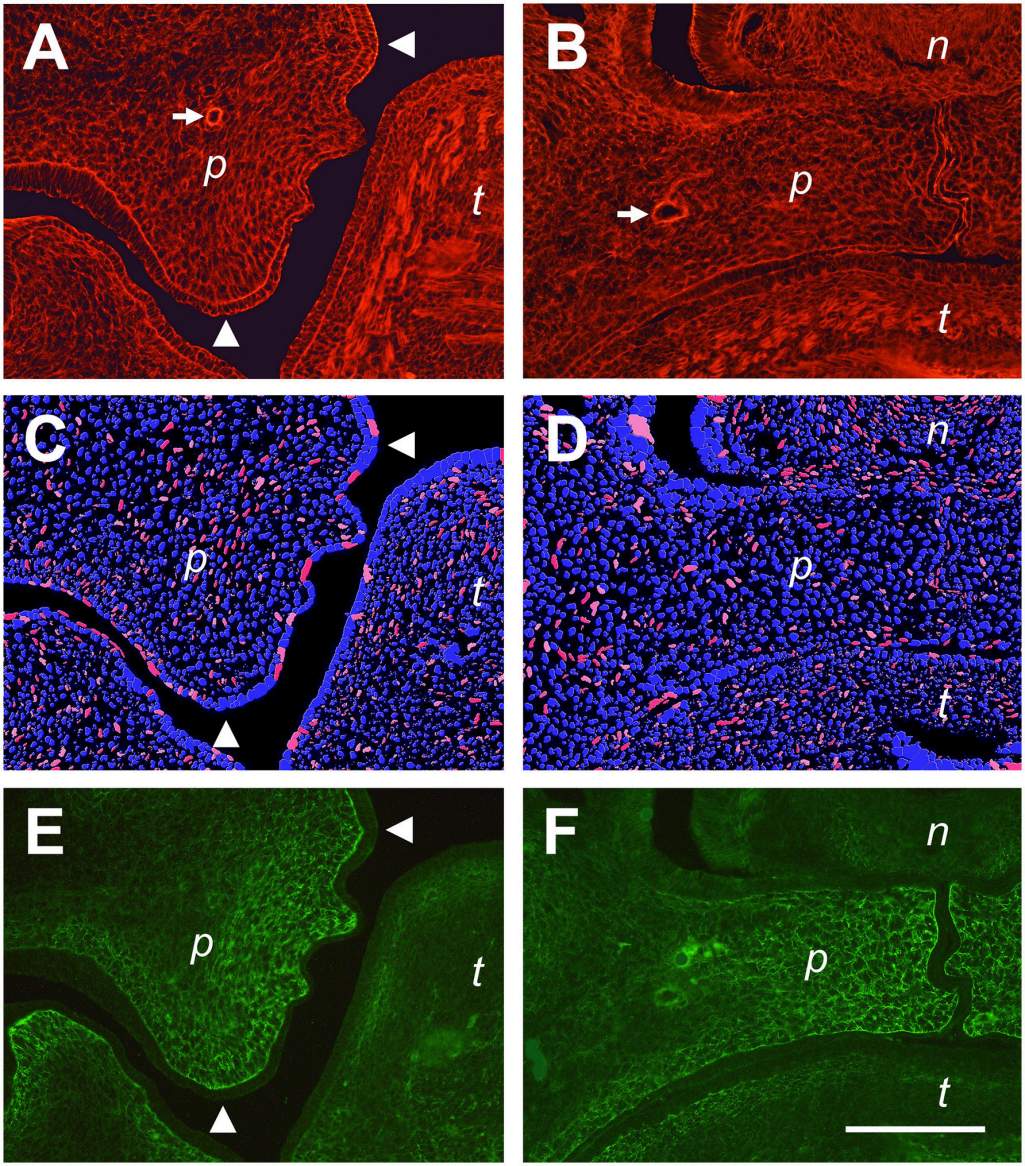
**Actin fiber and nuclear orientation in the mesenchyme of the posterior palate during elevation**. Representative sections through E14.5 palatal shelves were triple-stained with phalloidin for actin **(A,B)**, DAPI for nuclei **(C,D)**, and antibody to tenascin-C **(E,F)**, respectively, and viewed in a fluorescence microscope. On the left **(A,C,E)**, a shelf just before elevation is shown, and on the right **(B,D,F)** an already elevated shelf from an embryo of the same litter. The images **(C,D)** were processed by ImageJ, such that all elongated nuclei with an aspect ratio above 2.5 are false-colored in red, those with 2.0–2.5 in pink. Roundish nuclei (aspect ratio < 2.0) are blue. Note that before elevation **(C)**, many elongated (red/pink) nuclei are present between the original tip and the medial protrusion of the elevating shelf (arrowheads in **A,C,E**), and that their direction parallels that of the actin **(A)** and ECM **(E)** meshwork. The palatine artery is marked with an arrow in **(A,B)**. Abbreviations: palatal shelf (p), tongue (t), nasal septum (n). Bar, 200 μm.

Fluorescently labeled phalloidin is a specific marker for actin stress fibers of cells in culture (Small et al., 1999), and can also be used to label the cytoskeletal F-actin network of embryonic tissues (Figures 7A,B). As a major component of the contractile apparatus, actin stress fibers are known to align with the major direction of strain within a cell (Burridge and Wittchen, 2013). In Figure 7A, a higher magnification of a E14.5 palatal shelf at the medial level just before elevation (c.f. Figure 6C) is shown after phalloidin labeling. Note that the F-actin network of mesenchymal cells is aligned within this shelf, pointing toward the original ventral tip and the newly forming lingual protrusion, and forming an arc between them. In already elevated shelves of the same stage, the cellular actin network appears less organized, and no preferred direction is apparent in the shelf proper (Figure 7B). We also determined, on the same sections, the deformation of DAPI-labeled cell nuclei in the palatal mesenchyme. Using ImageJ, the ellipticity of stained nuclei was measured, and nuclei with an aspect ratio of >2.5 were labeled in red. As can be seen in Figure 7C, elongated (red) nuclei are enriched in, and align with, the arc-like F-actin network that is visible between the ventral and the lingual tips of this shelf before elevation. In contrast, elongated nuclei appear sparse and have no preferred direction in the elevated shelf (Figure 7D); they are more frequent in lateral maxillary mesenchyme. Note further that antibody to tenascin-C labels an ECM meshwork that lies parallel to the actin arc and the elongated nuclei in elevating shelves (Figure 7E). After elevation, the tenascin-C positive ECM has moved toward the midline as seen before, and the ECM fibrils appear randomly arranged (Figure 7F). These observations suggest that tensile stress builds up in the palatal shelves before elevation, which is released after the shelves have reached the horizontal position.

## Discussion

### Remodeling of palatal shelf mesenchyme during elevation revealed by ECM expression patterns

In the present study, we took advantage of the differential expression patterns of certain ECM genes to monitor tissue rearrangements during palatal shelf elevation. After screening over 20 ECM genes by *in situ* hybridization before and after elevation, we identified a few that exhibited expression patterns restricted to specific areas of the shelf mesenchyme or epithelium. Genes for ECM components are strongly expressed, and the turnover of their mRNAs is relatively slow (Eckes et al., 1996). We therefore argue that the observed changes in ECM expression patterns reflect morphogenetic rearrangements of palatal shelf tissue, rather than alterations in the rate of gene transcription. In the case of tenascin-C, *in situ* hybridization results were further confirmed by antibody stainings. This makes it even more unlikely that the observed dynamic changes in tenascin-C patterns during shelf elevation are due to rapid alterations in its synthesis.

Our results confirm the findings of Jin et al. (2010) concerning palatal shelf remodeling during elevation (see below). In addition, they extend earlier studies by providing more detailed information on the reshaping and medial translocation of the lingual shelf mesenchyme during this morphogenetic process. A further observation is noteworthy: During elevation, epithelial folds form dorsolaterally from the palatal shelves; these folds mark the lateral edges of the nasal cavity. Interestingly, the mesenchyme surrounding these folds express little or no periostin, which before elevation is a marker for the entire shelf proper. This suggests that at the epithelial folds, some originally nasal mesenchyme might be pulled into the elevating shelves. We have shown before that the epithelial folds are characterized by high expression of *Mmp2* mRNA and gelatinase activity, which presumably is needed there to avoid closure of the nasal cavity (Gkantidis et al., 2012). From our results, these specialized structures might originate from tissue outside the original shelf proper.

The mechanism of shelf elevation during secondary palate formation in mammals has been disputed (Brinkley and Vickerman, 1979; Jin et al., 2010; Bush and Jiang, 2012). Early reports favored a simple flipping movement of the vertical palatal shelves into a horizontal position (Coleman, 1965), but it was soon postulated that posteriorly the palatal shelves reorganize differently. From histological observations, it appeared that their lingual/medial aspect bulged out whereas their original ventral tip retracted (Brinkley and Vickerman, 1979). This process requires extensive remodeling of the entire shelf, but the underlying mechanism remained obscure. Recently, Jin et al. (2010) found that matrix metalloproteinase *Mmp13*, a specific marker for midline epithelial cells, was expressed in still vertical E14.5 shelves posteriorly not at their tip but on their lingual/medial aspect, whereas anteriorly the *Mmp13* signal extended into the shelf tip. The authors concluded that only anteriorly, the original tip epithelium will form the midline of elevated palatal shelves, whereas in middle and posterior regions, the midline epithelial cells are derived from the lingual epithelium of vertical shelves (Jin et al., 2010). These findings were in agreement with the earlier hypothesis that upon elevation, shelves undergo a flipping motion anteriorly, and a reshaping in middle and posterior regions.

(Jin et al., 2010) also showed that the mesenchymal transcription factor *Goosecoid* was expressed around the midline in E14.5 embryos, but in the lingual rather than the tip mesenchyme of non-elevated shelves of the same stage. This provided the first indication for large scale reorganization of pre-existing mesenchyme during shelf elevation, rather than for differential cell growth as it has been suggested (Brinkley and Bookstein, 1986; Sasaki et al., 2004). In any case, the rapid and often asynchronous time course of shelf elevation (Yu and Ornitz, 2011), as well as our present results, strongly argue against differential cell growth as a mechanism for palatal shelf remodeling.

### Actin-based cellular contractility combined with differential ECM stiffness as a possible mechanism for shelf remodeling during elevation

Cells attach to the ECM via integrins, which in turn are linked to the intracellular actin cytoskeleton. Embryonic cells and their ECM thereby form a mechanical continuum, in which actin-generated tensile stresses are transmitted from one cell to the next (Wozniak and Chen, 2009). Stresses align the intracellular F-actin, and also reorient and organize extracellular ECM fibers (Burridge and Wittchen, 2013). Moreover, cell nuclei are mechanically connected to the actin cytoskeleton and deformed by tensile stresses that pull on the cell (Brosig et al., 2010). Thus, preferred orientations of the interconnected actin-integrin-ECM meshwork, together with elongated cell nuclei, reflect vectors of tensile stress that act within embryonic mesenchyme. The fungal toxin phalloidin specifically binds to cellular F-actin (Small et al., 1999). Tenascin-C is known to decorate ECM fibrils in embryonic tissues (Chiquet-Ehrismann and Chiquet, 2003). We used these two tools, together with measurements of the ellipticity of cell nuclei, to determine whether tensile stresses might occur in palatal shelves during their elevation. Our results clearly indicate that in the medial and posterior parts of the elevating shelf, the mesenchyme is tensed between the original tip and the newly forming lingual/medial protrusion. It is more than likely that such tensile stress is generated by F-actin dependent contraction of the shelf mesenchymal cells themselves, and that it is transmitted onto the ECM in which they are embedded.

Despite of several hypotheses that have been put forward over the years, the forces driving palatal shelf elevation were essentially unknown. Walker and Fraser (1956) first postulated an “internal shelf force.” A popular hypothesis, still cited in recent reviews (Gritli-Linde, 2007), postulated that a cushion of hyaluronic acid in the “hinge” region of the palatal shelves provides a swelling force by generating osmotic pressure, which pushes up the shelf (Brinkley and Morris-Wiman, 1987). This idea was based on the presence of hyaluronidase-sensitive material in the respective region of the palatal shelf (Brinkley and Morris-Wiman, 1987), and on the fact that a glycosaminoglycan synthesis inhibitor caused cleft palate in mice (Brinkley and Vickerman, 1982). However, the main effect of the inhibitor was to cause a substantial shrinkage in shelf size compared to controls (Brinkley and Vickerman, 1982). Therefore, the inhibitor was likely to cause its damage long before shelf elevation occurred. In essence, there is no direct evidence that hyaluronic acid or other glycosaminoglycans are involved in generating the forces for shelf elevation.

Actin-based cellular contractility is well recognized for driving diverse morphogenetic movements during embryogenesis of both vertebrate and non-vertebrate species (Wozniak and Chen, 2009). Compared to alternatives such as differential cell growth or accumulation of ECM, this mechanism has a large advantage for the involved cells. Namely, they can control it rapidly and precisely in time and space, by activating the intracellular RhoA/ROCK signaling pathway that triggers actomyosin contraction (Burridge and Wittchen, 2013). Surprisingly, although actin contractility has been suggested as a mechanism for palatal shelf elevation early on (Lessard et al., 1974), this idea has apparently not been followed up in more recent years, but is now strongly supported by our current findings.

An actin-based mechanism for palatal shelf reorganization by no means implies that ECM itself has no function in the process. ECM sustains and counteracts forces generated by cellular contractility, and its local stiffness determines to which extent embedded cells are able to deform the tissue (Shawky and Davidson, 2015). Thus, if palatal shelf elevation is driven by cellular actin contraction, differences in ECM composition and stiffness within the shelf might have substantial effects on reshaping of the tissue. Moreover, specific ECM components can modulate cell adhesion, and thereby affect cell contractility. For example, tenascin-C inhibits RhoA/ROCK signaling (and thus actomyosin contraction) by interfering with cell adhesion to fibronectin (Midwood and Schwarzbauer, 2002). It can therefore be speculated that cell contraction is diminished in tenascin-C rich regions within the palatal mesenchyme. Future studies involving a careful mapping of F-actin dynamics, as well as of ECM composition and stiffness, within elevating palatal shelves will be required to fully understand this complex morphogenetic process.

## Author contributions

MC, SB, TM, and CK contributed to the conception and design of the work; MC, SB, and MA were responsible for data acquisition, analysis, and interpretation; MC drafted the manuscript; SB, MA, TM, and CK critically revised the manuscript; MC, SB, MA, TM, and CK approved the manuscript; MC, SB, MA, TM, and CK agreed to be accountable for all aspects of the work.

## Funding

MC, TM, and CK were supported by grant 31003A_146825 from the Swiss National Science foundation.

### Conflict of interest statement

The authors declare that the research was conducted in the absence of any commercial or financial relationships that could be construed as a potential conflict of interest.
